# Quadruple Semitendinosus Graft Construct With Double Cortical Suspensory Fixation for Anterior Cruciate Ligament Reconstruction: A Biomechanical Study

**DOI:** 10.1038/s41598-018-30931-7

**Published:** 2018-08-27

**Authors:** Carla Alexandra Madaíl, Maria de Fátima Vaz, Pedro Miguel Amaral, José Guimarães Consciência, Alcindo Lucas Silva

**Affiliations:** 1 Orthopedics and Trauma Department of Hospital de S. Francisco Xavier, Centro Hospitalar de Lisboa Ocidental Estrada Forte do Alto do Duque, 1449-005 Lisbon, Portugal; 2 Hospital Cuf Infante Santo, Travessa do Castro, 3, 1350-000 Lisbon, Portugal; 3 Nova Medical School, Universidade Nova de Lisboa, Campo Martires da Pátria 130, 1169-056 Lisbon, Portugal; 4 IDMEC, Instituto Superior Técnico, Universidade de Lisboa, Lisboa, Portugal; 5 Instituto Superior Técnico, Departamento de Engenharia Mecânica Av. Rovisco Pais, 1049-001 Lisbon, Portugal; 6 Hospital da Luz Arrábida, Praceta de Henrique Moreira 150, 4400-346 Vila Nova de Gaia, Porto Portugal

## Abstract

The purpose of this study was to evaluate the biomechanical properties of a graft construct with quadrupled Semitendinosus and two cortical buttons with adjustable loops concerning elongation, stiffness and resistance. A total of 15 fresh human cadaveric semitendinosus tendons were quadrupled over the two adjustable loops and stitched at the tibial tip with a cerclage type suture. They underwent pre-tensioning at 300 N for 2 minutes followed by cyclic loading (1000 cycles between 50–250 N) and finally a load-to-failure test. Statistical analysis was performed using SPSS Statistics software and groups were compared using a paired t-test, with a significance level set at α* = *0.05. Graft construct elongation after pre-tensioning at 300 N was 12.8 mm (9.3 mm–16.5 mm) and mean cyclic elongation 0.4 mm (0.2 mm–0.9 mm), considered significant (p < 0,001). The resistance and stiffness values were respectively 849.46 N (649.30 N-1027.90 N) and 221.49 N (178,30 N – 276.10 N). Quadruple ST graft construct using two cortical buttons and adjustable loops showed a high stiffness and resistance with a very low elongation after cycling.

## Introduction

Anterior cruciate ligament (ACL) reconstruction has become less invasive, tending to preserve bone stock as well as tendons and to improve fixation^1^.

Although a significant number of potential grafts for ACL reconstruction have been reported, the hamstring autografts are among the most commonly used.

The most common preparation technique using hamstring is the doubled semitendinosus (ST) and gracilis tendons (G). However, to decrease the morbidity associated to hamstring harvesting, a quadrupled ST graft construct has been developed in the last few years^2,3^. This technique has the advantage of preserving the gracilis, potentially improving postoperative hamstring strength^4^, besides providing a larger diameter than ST-G graft.

Recently, Silva *et al*.^5^ published a new quadruple ST graft construct using two cortical button and adjustable loops. The purpose of this study was to evaluate its biomechanical properties.

It was hypothesized that this quadruple ST graft construct with double cortical suspension provides a higher stiffness and resistance fixation along with a lower elongation after cycling.

## Materials and Methods

### Specimen Preparation

The study, in compliance with the Helsinki Declaration, was approved by the institutional ethics committee (DIFD – Department of Investigation, Formation and Documentation), South Section and Board of Directors of the National Institute of Legal Medicine and Forensic Sciences. It was confirmed that all the cadaver bodies used, were not included in the National Register of Non Donors (RENNDA form), which avoids the need for informed consent form.

A total of 15 fresh human cadaver semitendinosus tendons were harvested (age: 21–62) at the first 36 hours after death time.

Grafts were wrapped in a wet dressing (NaCl 0.9%) on sterile boxes, stored at 10 °C while waiting for the biomechanical analysis, performed up to one hour after harvesting. Tendons length was defined as 240 mm in order to achieve a constant folded graft length of approximately 60 mm.

### Graft Preparation

Both free ends of the semitendinosus were stitched over a length of 30 mm, using No. 2 ExpressBraid™ suture (Zimmer Biomet, Warsaw, IN). Two adjustable-length loop cortical button devices were used: ToggleLoc™ (Zimmer Biomet, Warsaw, IN), for the femur, and ToggleLoc XL Inline™ (Zimmer Biomet, Warsaw, IN), for the tibia. The tendon graft was symmetrically folded over the tibial cortical button loop and the doubled graft was passed through the femoral cortical button loop and once again symmetrically folded. Both grafts free ends are tied together with 3 knots over the tibial button loop. Then, the same sutures are tied over the graft itself using 4 knots^5^.

Finally, the graft is reinforced to the tibial side with a cerclage suture and a buried knot^6^.

To simulate the common tunnel lengths on the femur and tibia we assumed a constant femoral side length of 40 mm (including graft’s 15 mm) and a tibial side length of 50 mm (including graft’s 20 mm), adjusting both ToggleLoc™ loops, in order to achieve these values.

### Biomechanical Testing

**Figure 1:**
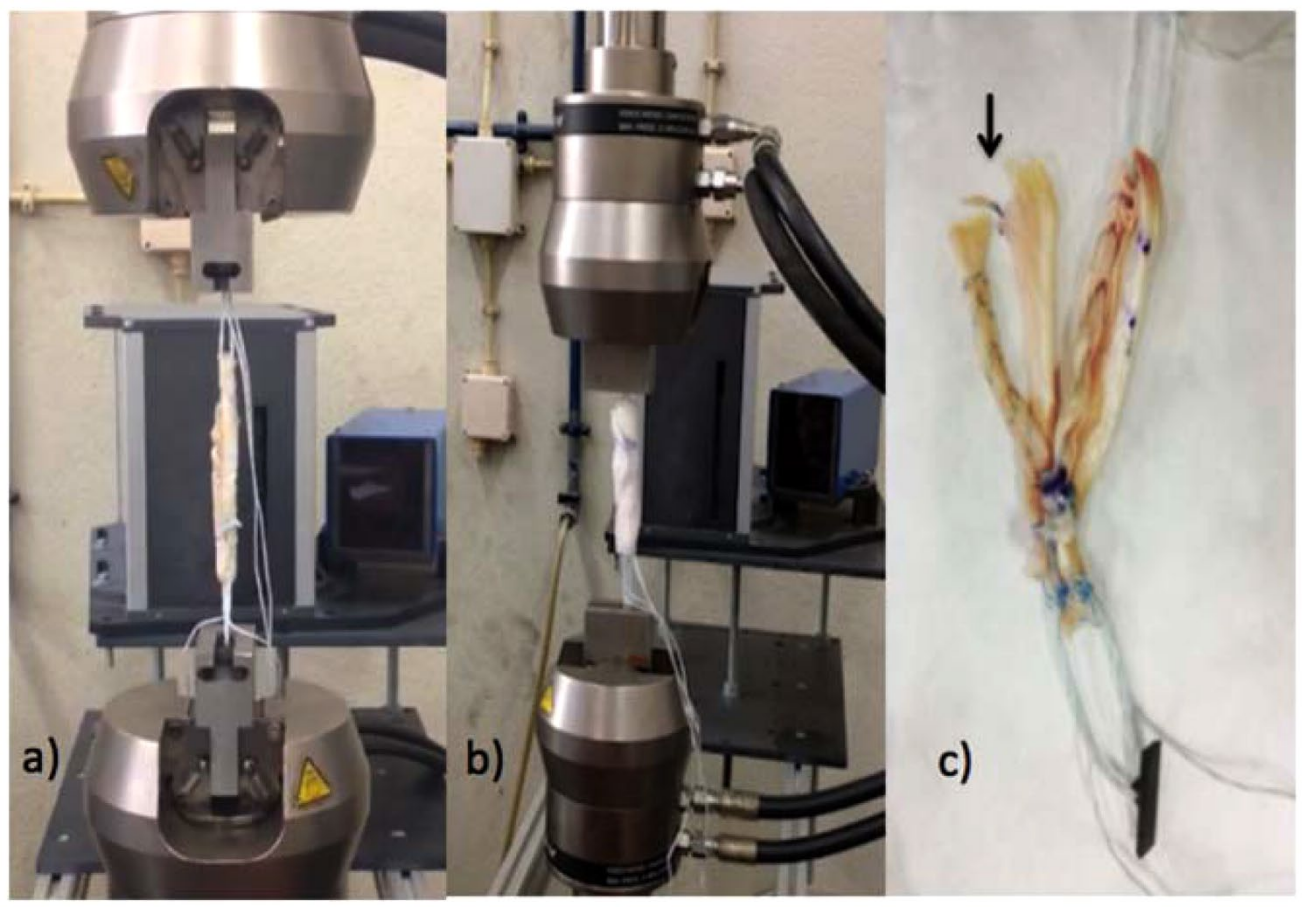
Test sequence (sample 904): (**a**) step 1; (**b**) step 7; (**c**) after test is completed.

Tests were conducted in a servo-hydraulic device (retrofitted Instron 8800 plus) with a loading cell of 10.0 kN (class 0.5). The graft construction ends (ToggleLoc™) were then placed in custom designed jigs which were rigidly fixed to the machine grips as shown in Fig. 1a.

The test protocol consisted in several steps from pretensioning to tensile test until failure finally took place (Table 1).

**Table 1:** Test Protocol.

step 1 - the graft was loaded from 0 to 300 N in 30 seconds;
step 2- the graft was preconditioned at 300 N for 2 min;
step 3 - there is an holding period of 30 seconds;
step 4 -the load decreases to 150 N in 10 seconds;
step 5 - the graft is submitted to cyclic loading between 50 and 250 N, for 1000 cycles at a frequency of 1 Hz;
step 6- there is an holding period of 30 seconds;
step 7 - the graft is submitted to a tensile test until failure.

Mean values with corresponding 95% confidence intervals (95% CI), standard deviation (SD), first quartile (Q1), median, third quartile (Q3), minimum (Min.) and maximum (Max.) values. C.E. - Clinical Equivalent, I.Op precond - intraoperative preconditioning, P.Op - postoperative. ^*^Difference variable.

Displacement and load were recorded by using the BlueHill Instron native software^7^. The elongation (millimeter) was determined as the difference between the displacement at a certain load and the initial load. For the load protocol, elongation during pre-tensioning (step 2) - Pre-tensioning elongation- and after 1000 cycles (step 5) - cycling elongation - were measured.

*Pre-tensioning elongation* represents the construct tensioning in traction table before graft implantation and *cycling elongation* represents the construct elongation after graft fixation followed by 1000 gait cycles.

A final tensile test (step 7) was done to evaluate both the ultimate load failure (ULF) and stiffness. Stiffness (N/mm) was then calculated as the load-elongation’s slope for the first part of the curve, i.e., until a load of 300 N. The final test set-up can be depicted in Fig. 1a.

The mechanism of failure was recorded (Fig. 1c).

### Statistical Analysis

Statistical analysis was performed using SPSS Statistics software, version 24 (IBM Corporation, Armonk, NY). The Shapiro–Wilk test was used to check whether the data had a normal distribution. Paired t-tests were used to compare groups of two values obtained by the same individuals in different circumstances. Data descriptions include means, 95% confidence intervals, standard deviations, first and third quartiles, median, maximum and minimum values. The significance level was set at α* = *0.05.

Test power calculation was obtained using R software (R Core Team (2017))^8,9^.

## Results

### Study power

The estimate of the correlation (ρ) between elongation after pre-tensioning at 300 N and after 1000 cycles, is given by the sample correlation (r) obtained from the sample, which was equal to 0.7.

Assuming that the standard deviations of each elongation (pre-tensioning at 300 N and after 1000 cycles) are equal, i.e., making the general assumption for the homogeneity of variance we consider that σ_300_ = σ_1000_ = σ where σ is estimated by the sample standard deviation s = 1.8 mm. We can then use the following expression to obtain the standard deviation for the differences (diff) in elongations between the pre-tensioning at 300 N and after 1000 cycles (Eq. 1).

1 $${\sigma }_{diff}=\sigma \sqrt{2(1-\rho )}$$

Consequently, we obtain an estimate of σ_diff_ equal to 1.394 mm.

In order to detect a difference of about 1 mm between elongations after 300 N and 1000 cycles, when using a sample of size 15 (n = 15) with 0.05 significance level and assuming that the standard deviation of the differences is 1.39 mm, the obtained power was 84%.(a)

### Graft

Mean quadruple ST graft final diameter was 9.7 mm (8–11 mm (Table 2)).

**Table 2:** Graft Characteristics.

N = 15	Mean 95% CI	SD	Q1	Median	Q3	Min.	Max.
Graft Length Before Pretensioning (mm)	59,67 (59.17; 60.16)	0.90	60	60	60	57	60
Graft Pretensioning Elongation (mm)	4,067 (3.46; 4.68)	1.10	3	4	5	3	7
Graft Diameter (mm)	9,267 (8.73; 9.80)	0.96	8	9	10	8	11

Mean values with corresponding 95% confidence intervals (95% CI), standard deviation (SD), first quartile (Q1), median, third quartile (Q3), minimum (Min.) and maximum (Max.) values.

### Pre-tensioning

Mean total graft construct’s elongation after pre-tensioning at 300 N was 12.8 mm (SD: 1.59 (Table 2)), with mean graft elongation of 4.1 mm (SD: 1.10 (Table 2)). The latest corresponding to 32% of total graft construct’s elongation.

### Cyclic loading

Mean cycling elongation (1000^th^ cycle) was 0.44 mm (SD: 0.24), considered significant (p < 0,001), comprising a confidence range between 0.31 and 0.57 mm (Table 3).

**Table 3:** Biomechanical testing summary.

N = 15	Mean 95% CI	SD	Q1	Median	Q3	Min.	Max.	C.E
Pre-tensioning elongation 300 N (mm)	12.81 (11.93; 13.69)	1.59	12.11	12.59	13.73	9.3	16.5	I.Op. precond
*Cycling elongation 1000 cycles(mm) – Elongation 300 N	0,4403 (0.31;0.57)	0.24	0.25	0.35	0.25	0.15	0.88	P.Op 1000 walking Cycles
Stiffness (N/mm)	221.50 (207.57; 235.43)	25.16	213.7	222.2	237.7	178.3	276.1	
Resistance (N) (ULF)	839.467 (776.02; 902.91)	114.57	740.33	840.2	915.7	649.3	1027.9	

Mean values with corresponding 95% confidence intervals (95% CI), standard deviation (SD), first quartile (Q1), median, third quartile (Q3), minimum (Min.) and maximum (Max.) values. C.E. - Clinical Equivalent, I.Op precond - intraoperative preconditioning, P.Op - postoperative. ^*^Difference variable.

**Figure 2:**
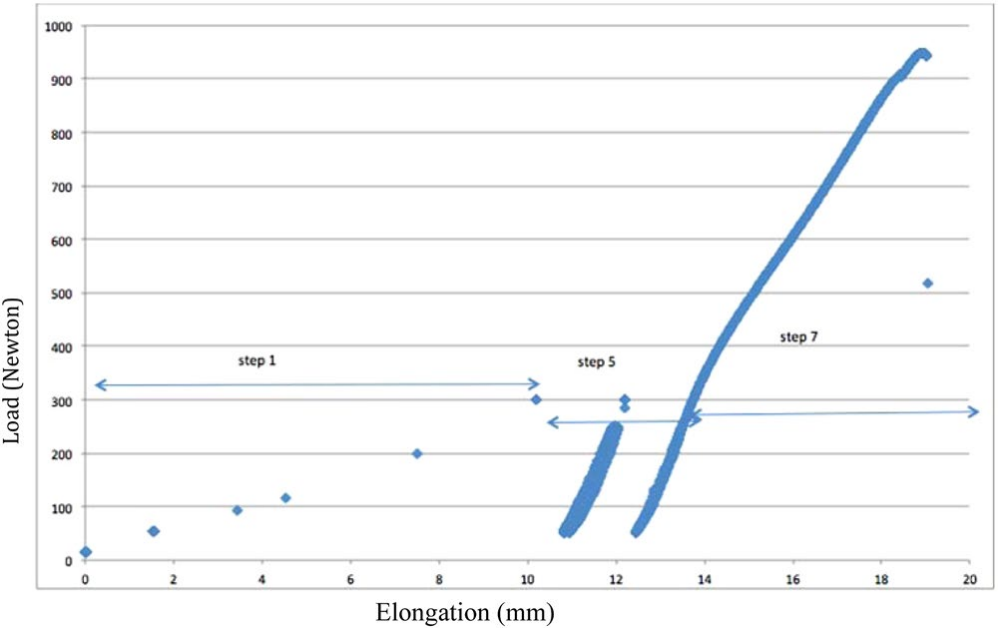
Load–elongation plot of a test (sample 961D) with the 7 steps. For the cyclic step only 3 cycles were shown (5, 30 and 1000^th^).

All specimens survived the cyclic loading testing (load-elongation depicted in Fig. 2).

### Load-to-failure test

Mean ULF and stiffness values were 849.46 N (SD:114.57) and 221.49 N/mm (SD:25.16), respectively (Table 3).

All failures occurred in the graft. In 13 out of 15, the rupture was at the femoral side folded graft (Fig. 1c). In the remaining two cases, one failed on the tibial side in the transition between the graft and suture and the other occurred after loosening of the 4 tied knots on the tibial side over the ST.

## Discussion

The most important finding of the present study was that a quadruple ST graft construct with two cortical suspension devices provided a higher stiffness and resistance fixation along with a lower elongation after cycling.

Elongation of the ACL graft construct should be kept to a minimum in order to avoid postoperative laxity and the risk of early ACL reconstruction failure.

With the development of adjustable-length loop cortical button devices, a new graft preparation technique using a cortical button graft fixation for the femur and tibia was introduced as described previously^10^. However, the effect of this preparation method on graft elongation is so far not known.

Proper pre-tensioning of graft construct is of extreme importance^11^ to allow a better accommodation of all the interfaces (graft, sutures and adjustable loops^12–14^) and thus avoiding a higher elongation after graft implantation. There is no consensus regarding how much load is actually needed for this pre-tensioning. Considering that the common walking loads acting on the graft are estimated to be around 298 N^15^, in the current study pre-tensioning was undertaken at 300 N load, in an attempt to reproduce physiological loads.

The elongation of the graft construct at this stage was 12.8 mm substantially higher than the one mentioned by Mayr *et al*.^16^. After pre-tensioning under 50 N, the elongation obtained in three different quadruple ST graft constructs ranged in-between 1.6 mm and 2.1 mm. Similar findings were reported by Petre^10^ referring a 2.45 mm elongation after pre-conditioning between 10 and 50 N. However, the used loads in those studies were 30 to 6 times lower (10–50 N) than the ones we applied.

In fact, greater elongation after pre-tensioning under high loads and low elongation after cycling, proves the importance of pre-tensioning in this kind of graft construct.

Another concern topic is elongation after cycling, corresponding to the elapsed time after graft implantation. Ideally, it should be lower than 3 mm difference (side to side), considered by some authors as the limit above which failure is usually due to laxity^17,18^.

In the present study, cumulative cyclic displacement determined after 1000 cycles was 0.4 mm and lower than the previous published data. According to Mayr *et al*.^16^ cyclic elongation after 1000 cycles ranged between 6.1 mm and 7.0 mm whereas Petre *et al*.^10^, mentioned higher cyclic elongation values (3.34 mm) and similar data were described by Barrow *et al*.^11^. These results are clearly higher than the ones obtained in our study (0.4 mm), suggesting that elongation after cycling is dependent on the selected pre-tensioning loads.

Stiffness and ultimate load failure of the studied graft construct were also evaluated. All the tested 15 constructs had mean values of stiffness (221.49 N/mm) and ULF (849.46 N) comparable or slightly higher than those published in the literature^16^.

There are several limitations to the current study.

First, the axial direction of the applied load, differs from clinical conditions^6,15,19^. This fact leads to higher stresses on the graft bending points over the adjustable loops. However, peak loads used in this study were similar to the forces experienced by the ACL during early rehabilitation^19,20^.

Secondly, it was not possible to hydrate the graft at the bending points (Fig. 1b), in opposition to the “*in vivo*” conditions where graft is permanently hydrated, increasing graft fragility and decreasing the ULF.

Finally, it is important to emphasize that this *in vitro* study focuses solely on graft construct biomechanics and does not include any biological factors.

Nonetheless, in this study, we used fresh human cadaver grafts, minimizing the bias that can be associated to frozen human or animal grafts.

The clinical relevance of this study comes from the fact that this graft construct technique may allow a safe rehabilitation with a low risk of elongation.

## Conclusion

Quadruple ST graft construct using two cortical buttons with adjustable loops, showed a higher stiffness and resistance with a very low elongation after cycling.

Pre-tensioning the graft construct under high loads, seems to prevent from additional significant displacement after cycling, assuming a critical role in its preparation.

## References

[1] Viola RW, Sterett WI, Newfield D, Steadman JR, Torry MR (2000). Internal and external tibial rotation strength after anterior cruciate ligament reconstruction using ipsi- lateral semitendinosus and gracilis tendon autografts. Am. J. Sports Med..

[2] Benea H (2014). Pain evaluation after all-inside anterior cruciate ligament reconstruction and short term functional results of a prospective randomized study. Knee..

[3] Hoher J (2000). Mechanical behavior of two hamstring graft constructs for reconstruction of the anterior cruciate ligament. J. Orthop. Res..

[4] Zamarra G, Fisher MB, Woo SL, Cerulli G (2010). Biomechanical evaluation of using one hamstrings tendon for ACL reconstruction: a human cadaveric study. Knee Surg. Sports Traumatol. Arthrosc..

[5] Silva A, Sampaio R (2015). Quadruple Semitendinosus Graft Construct and Suspensory Button Fixation for Anterior Cruciate Ligament Reconstruction. Arthroscopy. Techniques.

[6] Lubowitz JH (2012). All-inside anterior cruciate ligament graft link: graft preparation technique. Arthrosc. Tech..

[7] Instron. Testing software. [accessed Jan 2018] Available from, http://www.instron.us/en-us/products/materials-testing software/bluehill-software.

[8] R Core Team. R. A language and environment for statistical computing. R Foundation for Statistical Computing, Vienna, Austria. [accessed Jan 2018] Available from, http://www.R-project.org/ (2013).

[9] Stephane Champely. pwr: Basic functions for power analysis. R package version 1. 1.1. [accessed Jan 2018] Available from: http://CRAN.R-project.org/package=pwr (2012).

[10] Petre BM (2013). Femoral cortical suspension devices for soft tissue anterior cruciate ligament reconstruction: a comparative biomechanical study. Am. J. Sports Med..

[11] Barrow AE, Pilia M, Guda T, Kadrmas WR, Burns TC (2014). Femoral suspension devices for anterior cruciate ligament reconstruction: do adjustable loops lengthen?. Am J Sports Med..

[12] Howard ME, Cawley PW, Losse GM, Johnston RB (1996). Bone-patellar tendon-bone grafts for anterior cruciate ligament reconstruction: the effects of graft pretensioning. Arthroscopy..

[13] Brown CH, Wilson DR, Hecker AT, Ferragamo M (2004). Graft-bone motion and tensile properties of hamstring and patellar tendon anterior cruciate ligament femoral graft fixation under cyclic loading. Arthroscopy..

[14] Johnson JS (2015). A biomechanical comparison of femoral cortical suspension devices for soft tissue anterior cruciate ligament reconstruction under high loads. Am. J. Sports Med..

[15] Shelburne K, Pandy M, Anderson F, Torry M (2004). Pattern of anterior cruciate ligament force in normal walking. Journal of Biomechanics..

[16] Mayr R (2016). Preparation techniques for all-inside ACL cortical button grafts: a biomechanical study. Knee Surg. Sports Traumatol. Arthrosc..

[17] Beynnon BD (2002). Anterior cruciate ligament replacement: comparison of bone-patellar tendon-bone grafts with two- strand hamstring grafts. A prospective, randomized study. J Bone Joint Surg Am..

[18] Weiler A, Schmeling A, Stohr I, Kaab MJ, Wagner M (2007). Primary versus single-stage revision anterior cruciate ligament reconstruction using autologous hamstring tendon grafts: a prospective matched-group analysis. Am. J. Sports Med..

[19] Toutoungi DE, Lu TW, Leardini A, Catani F, O’Connor JJ (2000). Cruciate ligament forces in the human knee during rehabilitation exercises. Clin. Biomech. (Bristol, Avon)..

[20] Harrington IJ (1976). A bioengineering analysis of force actions at the knee in normal and pathological gait. Biomed Eng..

